# Gene Expression Changes Associated with Dedifferentiation in Liposarcoma Predict Overall Survival

**DOI:** 10.3390/cancers13123049

**Published:** 2021-06-18

**Authors:** Nicholas Brian Shannon, Qiu Xuan Tan, Joey Wee-Shan Tan, Josephine Hendrikson, Wai Har Ng, Gillian Ng, Ying Liu, Grace Hwei Ching Tan, Jolene Si Min Wong, Khee Chee Soo, Melissa Ching Ching Teo, Claramae Shulyn Chia, Chin-Ann Johnny Ong

**Affiliations:** 1 Department of Sarcoma, Peritoneal and Rare Tumours (SPRinT), Division of Surgery and Surgical Oncology, National Cancer Centre Singapore, Singapore 169610, Singapore; nicholas.shannon@mohh.com.sg (N.B.S.); qiu.xuan@nccs.com.sg (Q.X.T.); joey.tan.w.s@nccs.com.sg (J.W.-S.T.); josephine.hendrikson@u.duke.nus.edu (J.H.); nmsnwh@nccs.com.sg (W.H.N.); gillian.ng.w.x@nccs.com.sg (G.N.); liu.ying@nccs.com.sg (Y.L.); grace.tan.h.c@singhealth.com.sg (G.H.C.T.); jolene.wong.s.m@singhealth.com.sg (J.S.M.W.); soo.khee.chee@singhealth.com.sg (K.C.S.); melissa.teo.c.c@singhealth.com.sg (M.C.C.T.); claramae.chia.s.l@singhealth.com.sg (C.S.C.); 2 Department of Sarcoma, Peritoneal and Rare Tumours (SPRinT), Division of Surgery and Surgical Oncology, Singapore General Hospital, Singapore 169608, Singapore; 3 Laboratory of Applied Human Genetics, Division of Medical Sciences, National Cancer Centre Singapore, Singapore 169610, Singapore; 4 SingHealth Duke-NUS Oncology Academic Clinical Program, Duke-NUS Medical School, Singapore 169857, Singapore; 5 Institute of Molecular and Cell Biology, A*STAR Research Entities, Singapore 138673, Singapore

**Keywords:** liposarcoma, dedifferentiation, well-differentiated, gene expression, prognosis

## Abstract

**Simple Summary:**

Previous studies have performed integrative analyses of genomic aberrations in soft tissue sarcomas. Utilising clinical information, groups have proposed nomograms for prediction of survival and recurrence in retroperitoneal sarcomas. Expanding on clinical nomogram prediction models with molecular classification of tumours may allow us to further identify clinical phenotypes within this heterogeneous group. We showed that a five-gene molecular prognostic panel can provide additional prognostic information in patients with retroperitoneal DDLS, independent of clinical features. A combined clinical and molecular prediction model may offer the best way to prognosticate patients for patient counselling and therapeutic decision making.

**Abstract:**

Up to 10% of well-differentiated liposarcoma (WDLS) progress to dedifferentiated liposarcoma (DDLS). We aimed to identify gene expression changes associated with dedifferentiation and whether these were informative of tumour biology of DDLS. We analysed datasets from the Gene Expression Omnibus (GEO, ID = GSE30929) database to identify differentially expressed genes between WDLS (*n* = 52) and DDLS (*n* = 39). We validated the signature on whole and laser-capture microdissected samples from patients with tumours consisting of mixed WDLS and DDLS components. A subset of this signature was applied to an independent dataset from The Cancer Genome Atlas (TCGA, *n* = 58 DDLS) database to segregate samples based on gene expression and compared for recurrence and overall survival (OS). A 15-gene signature consisting of genes with increased expression in DDLS compared to WDLS was generated. This signature segregated WDLS and DDLS samples from patients with mixed component tumours and across multiple recurrences. A further subset of this signature, consisting of five genes (AQP7, ACACB, FZD4, GPD1, LEP), segregated DDLS in a TCGA cohort with a significant difference in OS (*p* = 0.019) and recurrence-free survival (RFS) (*p* = 0.061). The five-gene model stratified DDLS into prognostic groups and outperformed clinical factors in existing models in retroperitoneal DDLS.

## 1. Introduction

Soft tissue sarcomas (STSs) are a heterogeneous disease that comprise approximately 1% of all malignancies [1]. Clinical management is focused on preventing and treating recurrence with early surgery and systemic chemotherapy. Systemic treatment choices are increasingly made considering histology and molecular pathways associated with the disease, largely based on the response to different classes of agents.

Compared to those of more common tumour types, the molecular data available for sarcomas are limited. In 2010, Barretina et al. [2] performed integrative analysis of mutation, copy number, and expression data, but the reported results were largely limited to genomic aberrations in subtypes of STS, with a focus on liposarcoma. A more recent study from the Cancer Genome Atlas Research Network (CGARN) [3] reported a comprehensive integrative analysis of 206 adults with STSs representing six major types, including liposarcoma, for which they identified potential prognostic grouping defined by copy number and methylation changes.

In liposarcoma, the most common variant of STS, local recurrence occurs in two-thirds of patients, with metastasis in 50% of patients [4]. In particular, recurrence is common, with retroperitoneal tumours occurring at rates of 90–100%. However, little is known about the molecular changes underlying recurrence and whether these represent a distinct molecular subtype present at primary presentation or further genetic changes that occur in residual cells, allowing them to reinitiate tumour growth either locally or at secondary sites. Another feature of liposarcomas is the role of dedifferentiation, which is well established as a time- and location-dependent phenomenon associated with increased aggressiveness [5]. The term dedifferentiated liposarcoma (DDLS) was first coined in 1979 by Evans, describing a well-differentiated liposarcoma (WDLS) component juxtaposed to areas of high-grade non-lipogenic sarcoma [6]. Although WDLS is associated with high rates of local recurrence (30–50%), it has little to no metastatic potential. The risk of dedifferentiation in WDLS is up to 10% [7], and these DDLSs, in addition to the high rate of local recurrence, have a metastatic rate of 15–30% [8].

In their 2011 paper, Gobble et al. [9] compared WDLS and DDLS and suggested that progression is driven by differential expression of critical cell cycle, transcription factor, cytoskeleton/microtubule assembly-related, and apoptotic control genes. However, they also highlighted similar comparisons between WDLS/DDLS and normal fat, suggesting that these pathways represent a continuation of the same underlying tumorigenic processes rather than a distinct process of dedifferentiation.

Utilising clinical information, several groups have proposed nomograms for the prediction of survival and recurrence in retroperitoneal sarcomas [10,11,12]. Following the success of the MSKCC nomogram [10], Dalal et al. [13] generated a more accurate liposarcoma-specific nomogram based on age, presentation status, primary site, histologic variant, tumour burden, and gross margin status. The improved prognostication of patients using such nomograms has a role in improved patient counselling as well as planning further treatment based on expected survival. Expanding on clinical nomogram prediction models with molecular classification of tumours may allow us to further identify distinct clinical phenotypes within this heterogeneous group, which may impact clinical decision making. Furthermore, the identification of such features may be useful in the development of targeted and personalised therapy.

Thus, the objective of this study was to utilise publicly available gene expression data to identify markers stratifying WDLS/DDLS, to validate this across patient recurrences and within heterogeneous samples, and then to explore the role of this information in stratifying patients into good and poor prognostic groups to further inform clinical decision making. 

## 2. Materials and Methods

All experiments were conducted in line with the relevant guidelines and regulations, as approved by the SingHealth Centralized Institutional Review Board (CIRB 2015/3019/F). Informed consent was obtained from all study participants. 

### 2.1. External Datasets

External datasets were obtained from the Gene Expression Omnibus (GEO) and The Cancer Genome Atlas (TCGA) databases (Table 1). GSE30929 [9] consists of 140 primary liposarcomas profiled on Affymetrix Human Genome U133A Arrays. The data utilised for this study consisted of normalised gene expression data. TCGA-SARC [3] consists of 259 sarcoma samples, including 58 DDLSs with extensive genomic characterisation, such as mutation, copy number, methylation, and RNA profiling. The data utilised for this study consisted of the Illumina HiSeq Level 3 RSEM normalised expression data.

### 2.2. Patient Samples and Laser-Capture Microdissection (LCM)

**Figure 1:**
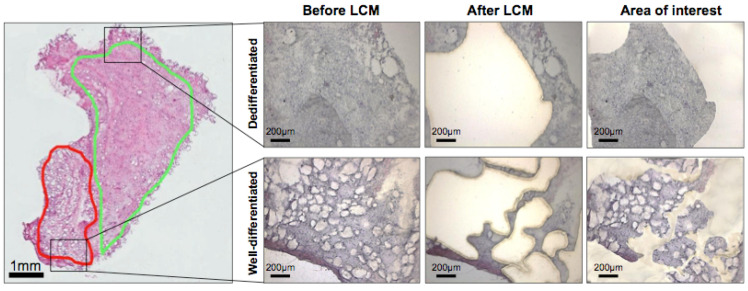
Representative sample of a liposarcoma with mixed well-differentiated liposarcoma (WDLS) and dedifferentiated liposarcoma (DDLS) components selected for laser-capture microdissection (LCM). Digitised haematoxylin and eosin (H&E) images were annotated by the clinical pathologist to identify well-differentiated (WD) (green) and dedifferentiated (DD) (red) regions. *LCM, laser-capture microdissection.* Scale bar: 1 mm (whole sample on left) or 200 µm (LCM samples on right).

Biopsies were systematically harvested. With liquid nitrogen, the harvested samples were snap frozen immediately upon resection to preserve good morphology and RNA integrity for histological assessment and transcriptomic analysis, respectively. The tissues of interest, namely the well-differentiated (WD) and dedifferentiated (DD) regions, were marked by a clinical pathologist on digitalised haematoxylin and eosin (H&E)-stained slides, as shown in Figure 1. Prior to LCM, the cryopreserved samples were sectioned at a thickness of 10 µm and prepared on PEN membrane slides to facilitate the microdissection process. Subsequently, the slides were subjected to the cresyl violet–eosin quick staining protocol, which provided good morphological resolution of the tissue samples while preserving the RNA integrity by reducing the tissue handling time. Using the pathologically annotated image as a reference, the areas of interest were identified and microdissected (Figure 1).

LCM was performed on frozen human liposarcoma samples to isolate two histological subtypes of the same tumour—WD and DD2010034—from three individuals with tumours consisting of both subtypes. An additional six whole samples from the same individuals were profiled, representing additional recurrences in the same patient (before or after the sample with mixed subtype). RNA was extracted using the Qiagen RNeasy Micro kit (cat no. 74004, Hilden, Germany) and amplified cDNA from the Affymetrix WT Pico Reagent Kit was profiled on Affymetrix GeneChip^®^ Human Gene 2.0 ST arrays (cat no. 902499, Massachusetts, MA, USA).

### 2.3. Data Analysis

Expression data for GSE30929 were downloaded from the GEO database, utilising GEOquery (v2.46.0) [14]. The data for WDLS and DDLS samples were utilised for further analysis. A list of genes that were differentially expressed between these two subtypes of liposarcoma was generated using Limma (3.34.1) [15], and the top 15 differentially expressed genes were assessed on the LCM/whole sample data.

Normalisation of LCM data was performed using the RMA normalisation procedure (RMA function from package oligo v1.42.0) [16,17,18,19]. Unsupervised clustering was performed utilising the 13 genes from the 15-gene signature, which mapped forward onto the dataset.

Expression data for the TCGA-SARC dataset were downloaded from the TCGA database utilising the RTCGA package (v1.8.0) [20]. The data for retroperitoneal DDLS were utilised for further analysis. Five genes were selected for further analysis based on which were the most prognostic for overall survival in the TCGA retroperitoneal DDLS data. The samples were segregated to good or poor prognostic groups utilising a model based on whether sample gene expression scores were above or below the median gene expression score across samples. Gene expression score was calculated as the total number of genes with low expression for each of the five genes in the final prognostic signature, giving a score from 0 to 5. The performance of the five-gene prognostic signature was assessed utilising Kaplan–Meier curves with the Cox proportional hazards model and receiver operating characteristic (ROC) curves. For univariate and multivariate analyses, samples were segregated based on two-year overall survival (OS) (*n* = 29). Factors were binarised utilising Youden’s index. Univariate analysis was performed utilising Fisher’s exact test and multivariate analysis of significant factors was performed using a factorial logistic regression model. All statistical analyses were performed using R (Version 3.4.2, open source) [21].

## 3. Results

### 3.1. Generation of the DDLS Signature

**Figure 2:**
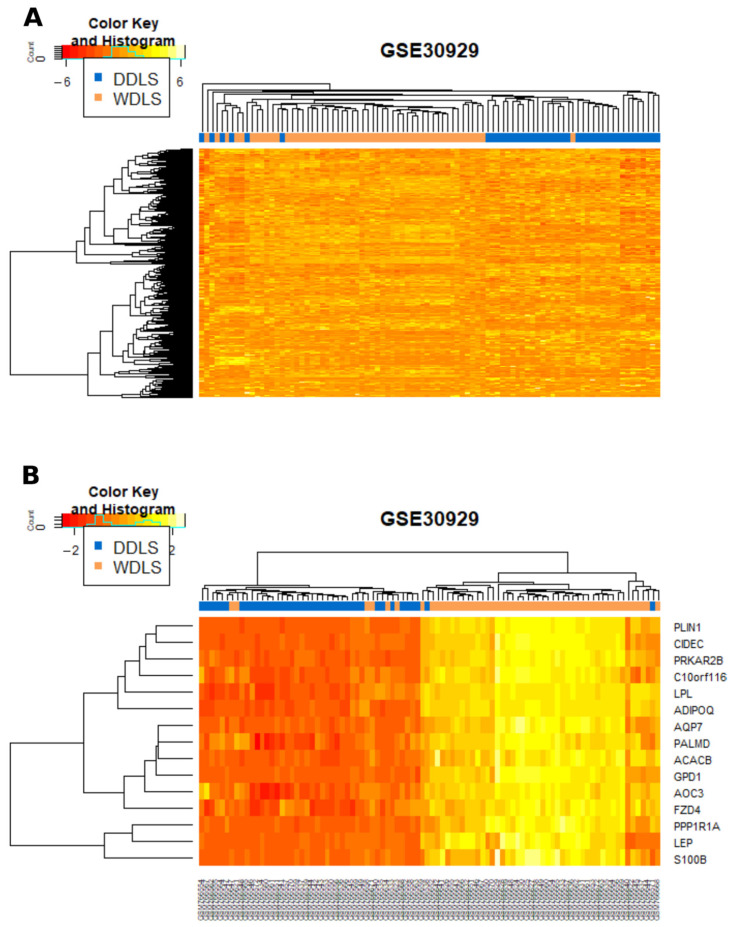
(**A**) Unsupervised clustering of dedifferentiated liposarcoma (DDLS) and well-differentiated liposarcoma (WDLS) from the GSE30929 dataset demonstrating segregation of the two subtypes. (**B**) Clustering of the GSE30929 dataset utilising the top 15 dysregulated genes between WDLS and DDLS. WDLS, well-differentiated liposarcoma; DDLS, dedifferentiated liposarcoma.

Unsupervised clustering of DDLS (*n* = 39) and WDLS (*n* = 52) from the Gobble dataset demonstrated that these two subtypes have distinct molecular profiles (Figure 2A). The top 15 differentially expressed genes between WDLS and DDLS were selected for further analysis of LCM/whole tissue samples. The signature was significantly enriched for genes involved in fatty acid metabolic processes (ADIPOQ, LPL, ACACB, LEP, PRKAR2B, *p* < 0.01). Clustering utilising these 15 genes demonstrated that this selective list retained the ability to classify samples into the two subtypes (Figure 2B).

### 3.2. Clustering of Matched DDLS and WDLS

**Figure 3:**
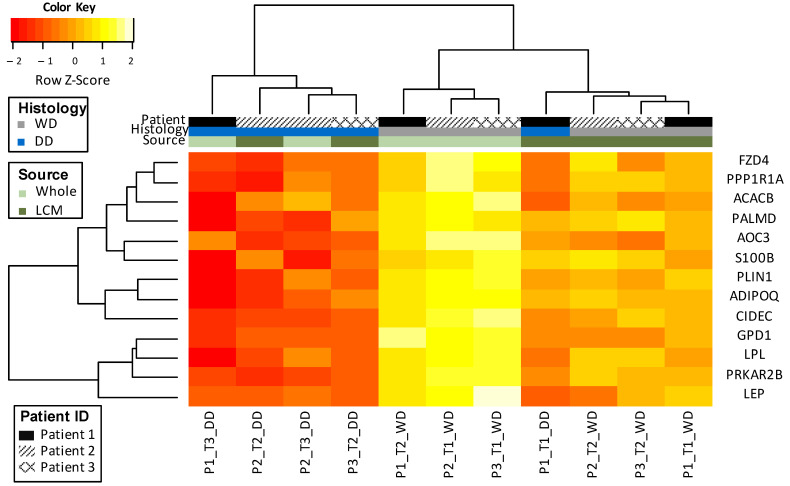
Segregation of liposarcoma samples by the 13-gene differentiation signature. WD, well-differentiated liposarcoma; DD, dedifferentiated liposarcoma; LCM, laser-capture microdissection.

We then validated the selected gene list in samples from patients with recurrent liposarcoma in both LCM samples of purified WDLS and DDLS from tumours with mixed components, as well as additional recurrences from the same patient. In the 15-gene signature, two genes were not present on the array. Hence, 13 genes that were mapped to the array were used to profile the LCM samples. The 13-gene signature accurately stratified liposarcoma subtypes across recurrences as well as from LCM samples from tumours containing a mixture of both subtypes. Interestingly, the 13-gene signature may further stratify WDLS based on whether this subtype was observed in a heterogeneous tumour (mixed WDLS/DDLS LCM sample) or a pure WDLS tumour (whole sample) (Figure 3).

### 3.3. Prognostic Significance in TCGA Dataset

**Figure 4:**
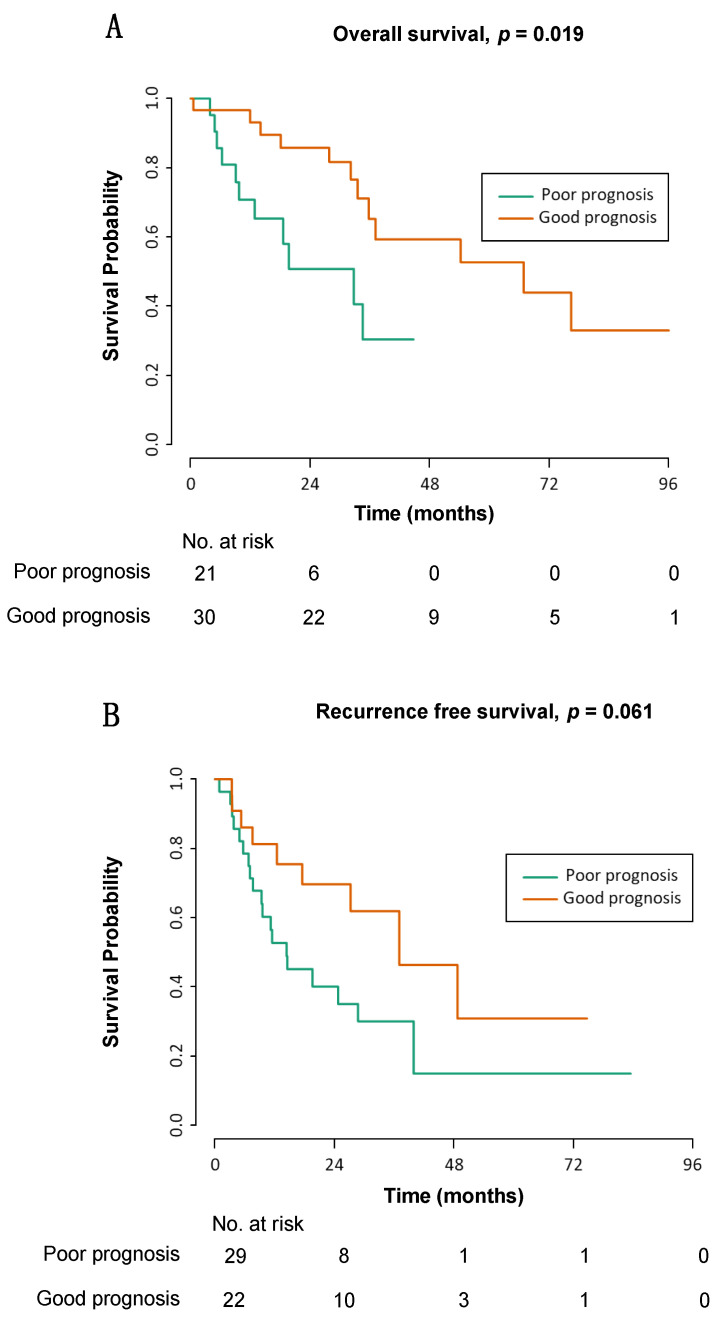
(**A**) Overall (*p* = 0.019) and (**B**) recurrence-free survival (*p* = 0.061) in retroperitoneal dedifferentiated liposarcoma (DDLS) as segregated by the five-gene prognostic signature.

Given the evidence that the signature can distinguish between DDLS and WDLS and that it may further subdivide these subtypes, we sought to assess its prognostic significance. We utilised a subset of five genes for a prognostic signature (AQP7, ACACB, FZD4, GPD1, LEP) applied to TCGA data. Utilising this signature, we could segregate retroperitoneal DDLS in the TCGA dataset into good and poor prognostic groups in OS (*p* = 0.019), almost reaching significance in recurrence-free survival (RFS) (*p* = 0.061) (Figure 4). The area under the ROC curve (AUC) for predicting two-year OS and RFS was 0.87 and 0.69, respectively, suggesting a good performance of this model for OS prediction.

### 3.4. Comparison to Clinical Information

Prediction of two-year survival time utilising the five-gene model (good or poor prognostic groups) was compared to clinical features of tumour size (reported width × length ≥ 460 cm^2^), margin status (negative or positive resection margins), recurrence (whether resected tumour represented recurrence or first presentation), and patient age (≥ 61 years) (Table 2). The univariate analysis, assessing the association between individual variables and the outcome measure (two-year survival) demonstrated the significance of both tumour size and the five-gene model (*p* < 0.05).

Subsequently, a multivariate analysis assessed the relationship between each of these and the outcome. Margin status was near statistical significance on univariate analysis (*p* = 0.056) only when considered as a gross margin, as it is typically used in nomogram prediction and was included in the multivariate analysis. Prediction using the five-gene model and gross margin status remained independent prognostic factors in multivariate analysis (*p* < 0.05), meaning they are prognostic independent of the other factors in the model.

### 3.5. Correlation with Normal Fat

To determine if there was history of the five genes being mutated or amplified in the most aggressive forms of LPS, we examined the mutation and copy number changes of these genes in the TCGA retroperitoneal sarcoma data.

ACACB was the only gene with any mutations, with 1 of 49 samples having a missense mutation (2%), and GPD1, LEP, and ACACB had copy number gains in 2 of 51 samples (4%). We also assessed the Human Protein Atlas (www.proteinatlas.org, accessed on 11 June 2021), which gives the protein and mRNA expression of genes across a variety of tissue types, including normal adipose (Supplementary Table S1).

## 4. Discussion

This study aimed to identify gene expression markers of dedifferentiation in liposarcoma, as well as to explore whether such markers could stratify patients into good and poor prognostic groups. We demonstrated that a modest signature of 13 genes, including those involved in fatty acid metabolic processes, can distinguish between DDLS and WDLS components of mixed liposarcomas. In addition, these markers could correctly stratify recurrences from the same patients into WDLS or DDLS. Furthermore, dysregulation of this gene signature was identified in WDLS samples from mixed tumours, suggesting that molecular changes associated with dedifferentiation may be detected before histopathological diagnosis, potentially allowing early prediction or diagnosis of such.

Furthermore, we demonstrated the ability of a five-gene dedifferentiation signature to stratify retroperitoneal DDLS into good and poor prognostic groups, and further showed that this prediction offers additional information beyond that provided by clinical information alone. This finding is complementary to work by other groups who developed nomograms utilising clinical data to predict recurrence and OS [22,23,24]. Potentially, a combination of both clinical and molecular information offers the best approach for the prognosis of patients as a clinical adjunct for more accurate patient counselling and improved identification of patients who may benefit from more aggressive treatment or adjuvant therapy for optimum disease control.

One limitation of the paper is the limited clinical follow-up data in the TCGA cohort. For this reason, we focused on the prediction of two-year survival to limit sample dropout (*n* = 29 from 51). The implications of this paper may also be limited by its generalisability. Previous groups have shown the benefit of population specificity; that is, a more accurate prediction is obtained for a liposarcoma-specific nomogram [13] compared to a generalisable sarcoma nomogram [10]. Thus, we streamlined our work to specifically assess retroperitoneal DDLS, as this group is associated with poor survival and heterogeneous outcomes. As such, more accurate prognostication is needed [25,26]. However, the significance of the results in WDLS and DDLS in the extremities are currently unknown. The specificity of this group may also limit the performance of clinical information, although the data suggest that current clinical nomograms are poorly discriminatory in retroperitoneal DDLS, which are typically larger than sarcomas in the extremities at presentation.

## 5. Conclusions

In conclusion, our study demonstrated that a five-gene molecular prognostic panel can offer added prognostic information in patients with retroperitoneal DDLS, independent of clinical features. This finding supports the concept that distinctive molecular features drive this disease phenotype. A combined clinical and molecular prediction model may offer the best way to assess patients for more accurate patient counselling and therapeutic decision making.

## Figures and Tables

**Table 1:** Transcriptomics datasets analysed as part of the study.

ID	First Author	Published	Samples; of Which Liposarcoma
GSE30929	Gobble	Cancer Res 2011 [9]	140; WDLS (*n* = 52), DDLS (*n* = 30), MLS (*n* = 17), RCLS (*n* = 12), PLS (*n* = 20)
TCGA-SARC	CGARN	Cell 2017 [3]	259; DDLS (*n* = 58)

Abbreviations: WDLS, well-differentiated liposarcoma; DDLS, dedifferentiated liposarcoma; MLS, myxoid liposarcoma; PLS, pleomorphic liposarcoma; RCLS, round cell liposarcoma; CGARN, Cancer Genome Atlas Research Network.

**Table 2:** Multivariate comparison of model prediction and clinical factors. W = width, L = length, R2 = gross positive margin, R1 = microscopically positive margin, R0 = negative margin.

Factors	Univariate		Multivariate	
	*p*-Value	OR	*p*-Value	OR
Size (W × L ≥ 460 cm^2^)	0.019	15.4	0.087	1.33
Margin status (R1/R2 vs. R0)	0.183	5.7	-	-
Margin status (R2 vs. R1/R0)	0.056	NA	0.034	2.1
Age (≥61 years)	0.711	1.6	-	-
Recurrence (recurrence vs. primary)	1.000	NA	-	-
Model prediction (poor vs. good prognosis)	<0.010	15.75	<0.01	2.0

## Data Availability

The data that support the findings of this study are available from the corresponding author upon reasonable request.

## Supplementary Materials

The following are available online at https://www.mdpi.com/article/10.3390/cancers13123049/s1, Table S1: Mutations and copy number changes in dedifferentiated liposarcoma in the TCGA cohort. Protein and mRNA expression of genes in normal adipose tissue as reported in the Human Protein Atlas (www.proteinatlas.org, accessed on 11 June 2021).
