# Supplementary material for: Gene Expression Changes Associated with Dedifferentiation in Liposarcoma Predict Overall Survival
Source: Cancers (Basel). 2021 Jun 18;13(12):3049. doi: 10.3390/cancers13123049 (PMC8235385; doi:10.3390/cancers13123049)
Supplement: Supplementary file 1 [file cancers-13-03049-s001.zip › cancers-1261701-supplementary.pdf]

Supplementary Material: Gene Expression Changes Associated with Dedifferentiation in Liposarcoma Predict Overall Survival

Nicholas Brian Shannon, Qiu Xuan Tan, Joey Wee-Shan Tan, Josephine Hendrikson, Wai Har Ng, Gillian Ng, Ying Liu, Grace Hwei Ching Tan, Jolene Si Min Wong, Khee Chee Soo, Melissa Ching Ching Teo, Claramae Shulyn Chia and Chin-Ann Johnny Ong

Table S1. Mutations and copy number changes in dedifferentiated liposarcoma in the TCGA cohort. Protein and mRNA expression of genes in normal adipose tissue as reported in the Human Protein Atlas (www.proteinatlas.org, accessed on 11 June 2021).

**Gene**	**DDLS Mutations (n = 49 Samples)**		**DDLS Copy Number Changes (n = 51 Samples)**		**Human Protein Atlas (Normal Adipose)**	
	Loss	Missense	Gain	Loss	Protein	mRNA
GPD1	0	0	2	1	High	High
ACACB	0	1	2	2	High	High
AQP7	0	0	1	2	NA	High
FZD4	0	0	1	2	Not detected	High
LEP	0	0	2	0	Low	High
